# Supplementation With *Lycium barbarum* Polysaccharides Reduce Obesity in High-Fat Diet-Fed Mice by Modulation of Gut Microbiota

**DOI:** 10.3389/fmicb.2021.719967

**Published:** 2021-08-26

**Authors:** Mei Yang, Yexin Yin, Fang Wang, Haihan Zhang, Xiaokang Ma, Yulong Yin, Bie Tan, Jiashun Chen

**Affiliations:** ^1^Animal Nutritional Genome and Germplasm Innovation Research Center, College of Animal Science and Technology, Hunan Agricultural University, Changsha, China; ^2^CAS Key Laboratory of Agro-ecological Processes in Subtropical Region, Institute of Subtropical Agriculture, Changsha, China

**Keywords:** *Lycium barbarum* polysaccharides, high-fat diet, gut microbiota, lipid metabolism, obesity

## Abstract

Lycium barbarum polysaccharides (LBPs) have been proved to prevent obesity and modulate gut microbiota. However, the underlying mechanisms of LBPs’ regulating lipid metabolism remain entirely unclear. Therefore, the purpose of this study was to determine whether LBPs are able to modulate the gut microbiota to prevent obesity. The results showed that oral administration of LBPs alleviated dyslipidemia by decreasing the serum levels of total triglycerides, total cholesterol, and low-density lipoprotein-cholesterol and elevating the high-density lipoprotein cholesterol in obese mice. Furthermore, LBP treatment decreased the number and size of adipocytes in epididymal adipose tissues and downregulated the expression of adipogenesis-related genes, including acetyl-CoA carboxylase 1, fatty acid synthase, stearoyl-CoA desaturase 1, sterol regulatory element-binding protein-1c, peroxisome proliferator-activated receptor γ, and CCAAT/enhancer-binding protein α. 16S rRNA gene sequencing analysis showed that LBPs increased the diversity of bacteria, reduced the *Firmicutes*/*Bacteroidetes* ratio, and improved the gut dysbiosis induced by a high-fat diet; for example, LBPs increased the production of short-chain fatty acid-producing bacteria *Lacticigenium*, *Lachnospiraceae_NK4A136_group*, and *Butyricicoccus*. LBPs treatment also increased the content of fecal short-chain fatty acids, including butyric acid. These findings illustrate that LBPs might be developed as a potential prebiotic to improve lipid metabolism and intestinal diseases.

## Introduction

Obesity is an important risk factor for many chronic diseases such as type II diabetes, cardiovascular and cerebrovascular diseases, cancer, and so on (Shin and Yoon, 2018), and which has become one of the top health problems in the world. It becomes a major challenge for modern societies to decrease the incidence of obesity and its associated diseases. The pathogenesis of obesity is complex that mainly involves genetic and environmental factors (Silventoinen et al., 2007). A wealth of evidence has demonstrated that the gut microbiota plays an important role in regulating nutrient acquisition and body weight, thus serving as a key factor to regulate obesity, and its associated disorders (Ridaura et al., 2013; Sanmiguel et al., 2015).

The gut microbiota primarily contains *Firmicutes*, *Bacteroidetes*, *Actinobacteria*, and *Proteobacteria* phyla, which are composed of more than 1,000 different bacterial species, but not all species are known to this date (Ley et al., 2005). A high-fat diet has been reported to reshape the gut microbiota, particularly by increasing the proportion of *Firmicutes* in relation to *Bacteroidetes*, which plays a significant role in the pathogenesis of obesity-induced metabolic diseases (Hildebrandt et al., 2009). Several studies provide scientific evidence that the gut microbiota is becoming a promising therapeutic target for dietary interventions to protect against obesity (Ojo et al., 2016; Zheng et al., 2018). In recent years, some plant-derived natural bioactive compounds, including polysaccharides, were reported to be helpful to reduce weight gain, and fat accumulation *via* the modulation of the gut microbiota (Shang et al., 2017; Sun et al., 2018).

Red-colored fruits of *Lycium barbarum* (Gouqizi, Fructus Lycii, or wolfberry) are used as traditional Chinese herbal medicine to promote health and longevity, and as a food supplement for 1,000 of years (Qian, 2019). *Lycium barbarum* polysaccharides (LBPs) are the main active constituents of *L. barbarum* fruits, which possess a variety of pharmacological effects, such as antioxidant, anti-stress, neuroprotective activities, anti-aging, antidiabetic activities, immune regulation, protection against liver damage, and reduction of blood glucose level (Luo et al., 2004; Ha et al., 2005). Recent studies have shown that LBPs play a vital role in regulating hepatic lipid metabolism (Jia et al., 2016). Furthermore, LBPs improve dyslipidemia, promotes energy expenditure, reduces body weight, and alleviates non-alcoholic steatohepatitis (Xiao et al., 2013, 2014). Dysfunction of hepatic energy signaling induced by a high-fat diet represents a key mechanism for hepatic insulin resistance and lipid accumulation associated with non-alcoholic fatty liver disease (Li et al., 2014). However, studies on the anti-obesogenic activity of LBPs and the related gut dysbiosis are limited. Our previous studies demonstrated that supplementation with LBPs in piglet diets stimulated the growth of beneficial gut bacteria and suppressed the growth of *Escherichia coli* (Chen et al., 2019). Although numerous health benefits of LBPs have been reported, their effects on the gut microbiota in animals with high fat-diet-induced dysbiosis are not known. Therefore, the objectives of the present study are to investigate the effects of LBPs on the gut microbiota, blood lipids, and genetic factors regarding lipid metabolism in high-fat diet-fed mice. The results will increase our understanding of how LBPs regulate gut microbiota to exert anti-obesogenic effects.

## Materials and Methods

### Animals and Dietary Treatments

All procedures were approved by the Animal Care and Use Committee of Hunan Agricultural University, People’s Republic of China (permit number: CACAHU 2020-0821). Three-week-old male ICR mice (specific pathogen-free) were purchased from Shanghai Laboratory Animal Central (Changsha, China). After a 1-week adaptation period, the mice were housed in a controlled environment (temperature: 23 ± 2°C, relative humidity: 50 ± 5%, and a 12-h light–dark cycle), with free access to food and drinking water during the experiment.

Thirty mice were randomly separated into three groups (*n* = 10), including the normal chow diet group (NC), the high-fat diet group (HFD), and the HFD-fed mice with the LBP group (HFD + LBPs). The NC group was fed with an NC diet (research diet D12450B, containing 10% kcal from fat, 3.85 total kcal/g, and Beijing Botai Hongda Biotechnology Co., Ltd.); mice in the HFD and HFD + LBP groups were fed an HFD (research diet D12492, containing 60% kcal from fat, and 5.24 total kcal/g) as model controls (Shang et al., 2017). Moreover, mice in the HFD + LBP group drank water containing 0.2% of LBPs from the beginning, and the other two groups received sterile water. In the current study, LBPs (high-performance liquid chromatography ≥ 60%) comprised D-mannose, L-rhamnose, D-glucose, D-galactosamine, and D-xylose, purchased from Xi’an ZeBang Biological Technology Co., Ltd. (Xi’an, China). Throughout the experiment, the body weight and food intake of mice were measured weekly for 10 weeks. Fecal samples were collected and stored at −80°C until further analysis. At the end of the experiment, all mice were fasted overnight and killed by cervical dislocation with sodium pentobarbital anesthesia, and all efforts were made to minimize suffering. After killing, blood, liver, epididymal adipose tissues, cecum, colon, and colon contents were collected for further analyses.

### Analysis of Biochemical Parameters in Blood and Liver Samples

Blood samples were collected from the orbital venous plexus of mice under anesthesia. The serum was obtained from blood samples with the centrifugation at 4,000 × *g* at 4°C for 10 min and stored at −80°C for further analysis. The levels of high-density lipoprotein cholesterol (HDL-C), low-density lipoprotein cholesterol (LDL-C), total cholesterol (TC), triacylglycerols (TG), and malondialdehyde (MDA) in serum and liver were monitored by the corresponding assay kits according to the manufacturer’s instructions (Nanjing Jiancheng Bioengineering Institute, China).

### Histology Analysis

The liver and epididymal adipose tissues were removed and fixed in 4% formaldehyde solution, after which the fixed tissues were paraffin-embedded and the liver and epididymal adipose tissues blocks were cut into 5-μm sections, and stained with hematoxylin and eosin.

### RNA Extraction and Gene Expression Analysis

Total RNA from the epididymal adipose tissues was isolated using Trizol reagent (Invitrogen, United States) and treated with DNase I (Promega Corporation, Germany) according to the manufacturer’s instructions. The complementary DNA (cDNA) was generated from total RNA according to the reverse transcription kit (TaKaRa Company, Dalian). The ABI 7900HT-polymerase chain reaction (PCR) instrument (ABI Biotechnology, United States) was used to amplify the samples by using SYBR-Green I dye (Molecular Probes, Eugene, OR, United States) and using the supporting software (Applied Biosystem, SDS2.3) for data analysis. The PCR primers sequences for the corresponding genes were listed in Supplementary Table 1. The PCR was performed in duplicate at 95°C for 3 min and subjected to 40 cycles of 95°C for 30 s, 55°C for 30 s, and 72°C for 45 s. The relative expression levels of target genes cDNA to β-actin cDNA were calculated as a ratio by the 2^–ΔΔCt^ formula.

### DNA Extraction and High Throughput Sequencing

Metagenomic DNA was extracted using the E.Z.N.A.^®^ Soil DNA Kit (Omega Bio-tek, United States) according to the manufacturer’s protocols. The V3–V4 regions of the cecal microbiota 16S rRNA gene were amplified by using specific primers of (338F: 5′ ACTCCTACGGGAGGCAGCAG-3′; 806R: 5′- GGACTACHVGGGTWTCTAAT-3′) by ABI GeneAmp PCR System 9700 (Applied Biosystems, Foster City, CA, United States) in triplicate. The PCR products were examined and purified from 2% agarose gels and purified using the AxyPrep DNA Gel Extraction Kit (Axygen Biosciences, United States) and quantified by QuantiFluor^TM^ -ST (Promega, United States). The purified amplicons were pooled in equimolar and ligated with 300-bp paired-end adapters by TruSeq^TM^ DNA Sample Prep Kit (Illumina, United States), then sequenced on an Illumina MiSeq platform (Illumina, United States) according to the standard protocols by Majorbio Bio-Pharm Technology Co., Ltd. (Shanghai, China).

### Bioinformatics Analysis

The raw sequencing data were quality trimmed and filtered by Trimmomatic and merged with FLASH according to the overlap sequences. The reads were truncated at any site accepting an average mass value less than 20 in a 50-bp sliding window (Wang W. et al., 2019). Operational taxonomic units (OTUs) were generated by clustering at 97% similarity using USEARCH v7.0,^1^ and chimeric sequences were identified and removed using UCHIME (Wang S. et al., 2018). The classification of each 16S rRNA gene sequence was performed by RDP Classifier v2.11^2^ according to the SILVA (Release132^3^) 16S rRNA database with a confidence threshold of 70%. Alpha diversity was analyzed using MOTHUR v1.30.2,^4^ and beta diversity was determined using QIIME. Alpha diversity analysis included Shannon and Chao index. Beta diversity included unweighted unifrac distances calculated with 10 times of subsampling, and these distances were visualized by principal coordinate analysis. To identify the dimensional gut bacteria and characterize the microbial differences between different groups, the linear discriminant analysis (LDA) effect size analysis was performed. The non-parametric factorial Kruskal–Wallis sum-rank test was applied to detect features that were significantly different between assigned taxa, and the LDA was used to quantify the effect size of each feature. A significance alpha value of less than 0.05 and an effect size threshold of 3 were used for this analysis.

### Analysis of Short-Chain Fatty Acids

The contents of colon and fecal samples were collected, and a mixture of supernatant fluid and 25% metaphosphoric acid solution (4: 1 ml) was prepared for the determination of SCFAs (acetic acid, butyric acid, propionic acid, and valeric acid). Samples were incubated at room temperature and centrifuged, and the supernatants were filtered by using 0.45-μm polytetrafluoroethylene syringe filters into chromatographic glass vials (Agilent Technologies). Gas chromatography was performed using an Agilent 6890 GC system with a flame ionizable detector and an automatic liquid sampler (Agilent Technologies, Santa Clara, CA, United States) as previously described (Chen et al., 2018).

### Statistical Analysis

One-way analysis of variance was used for statistical analysis using SPSS 25.0 software. Any differences among treatments were then compared using the Duncan comparison range tests. The experimental data are expressed as the means ± SEM; *P* < 0.05 among different groups were considered statistically significant.

## Results

### Animal Weight and Food Intake

**FIGURE 1:**
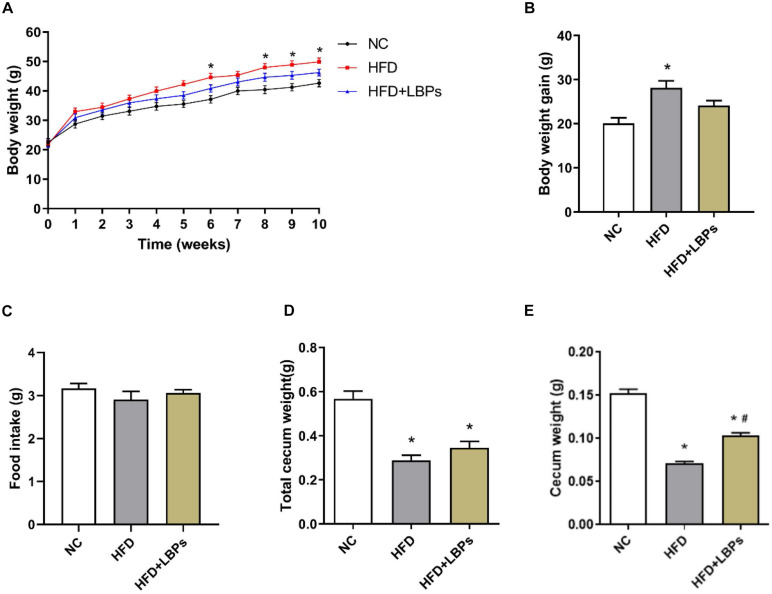
Effects of *Lycium barbarum* polysaccharides (LBPs) on **(A)** body weight, **(B)** weight gain, **(C)** food intake, **(D)** total cecum, and **(E)** cecum tissues in HFD-fed mice. *P < 0.05 vs. NC group and ^ #^*P* < 0.05 vs. HFD group.

The HFD group exhibited a 14% higher final body weight as compared with the NC group (Figure 1A) (*P* < 0.05). The HFD-fed mice treated with LBPs reduced body weight by 7% compared with the HFD group (Figure 1A) (*P* > 0.05). LBP supplementation slightly decreased the body weight gain in mice with HFD feeding (Figure 1B) (*P* > 0.05). Supplementation with LBPs has no significant effect on food intake in HFD-fed mice (Figure 1C) (*P* > 0.05). The weight of total cecum and cecum in the HFD group was lower than that of the NC group (Figures 1D,E) (*P* < 0.05). In addition, LBP treatment improved the weight of the cecum in the HFD group (Figure 1E) (*P* < 0.05).

### Serum and Liver Lipid Content

**FIGURE 2:**
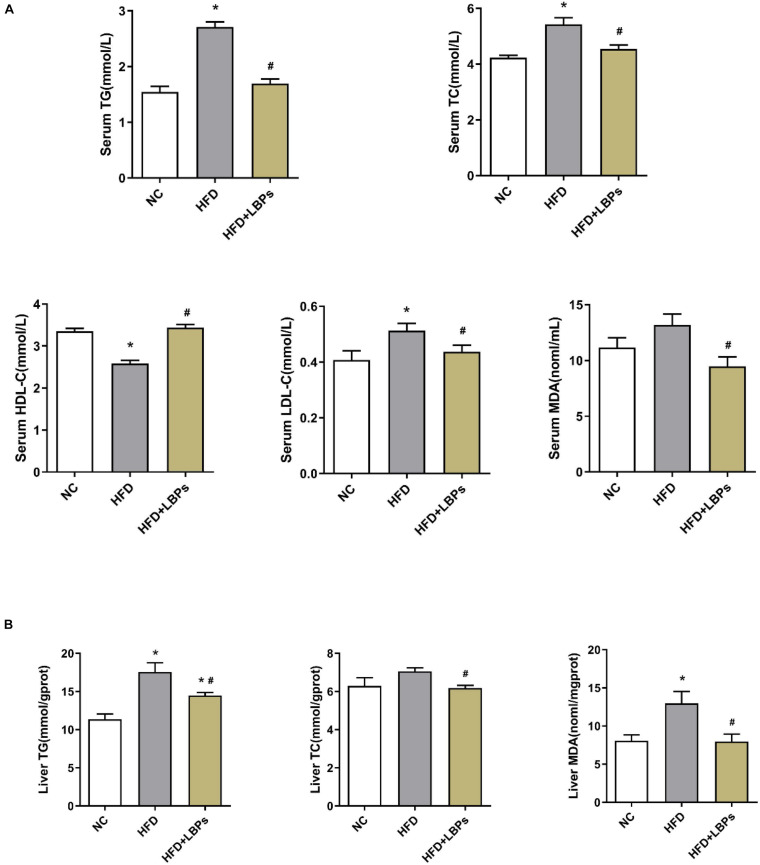
Effects of *Lycium barbarum* polysaccharides (LBPs) on **(A)** serum and **(B)** liver lipids profile in HFD-fed mice. *P < 0.05 vs. NC group and ^#^*P* < 0.05 vs. HFD group.

The consumption of dietary fat induces anomalous changes in lipid content, including TG, TC, HDL-C, and LDL-C. Compared with the NC group, HFD-fed mice had reduced HDL-C levels along with increased TG, and TC levels in serum (*P* < 0.05). Supplementation with LBPs increased HDL-C level and reduced the levels of TG, TC, and MDA in HFD-fed mice (Figure 2A) (*P* < 0.05). However, the TG, TC, and MDA contents in the liver were decreased after LBP administration in comparison with the HFD group (Figure 2B) (*P* < 0.05).

### Lipid Accumulation and Metabolism in Liver and Epididymal Adipose Tissues

**FIGURE 3:**
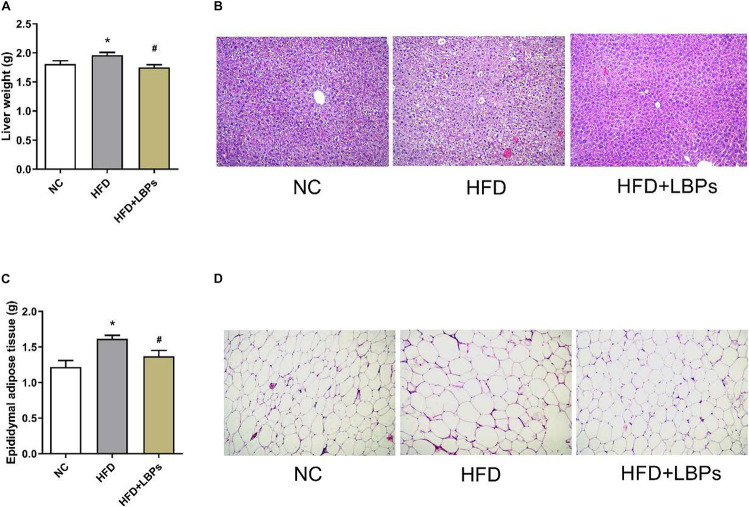
Effects of LBPs on fat accumulation in liver and adipose tissues in HFD-fed mice. **(A)** Liver weight, **(B)** histology analysis of liver, **(C)** epididymal adipose tissues weight, and **(D)** histology analysis of epididymal adipose tissue. *P < 0.05 vs. NC group and ^#^*P* < 0.05 vs. HFD group.

Compared with the NC group, the HFD group increased the weight of liver, and epididymal adipose tissues. Treatment with LBPs reduced this parameter in the HFD + LBP group compared with the HFD group (Figures 3A–C) (*P* < 0.05). Histology analysis revealed that fat accumulation occurred in the HFD group. LBP administration decreased the fat accumulation, and the size and number of adipocytes in adipose tissues were near to the NC group (Figures 3B–D).

### Adipogenesis-Related Gene Expression in the Epididymal Adipose Tissues

**FIGURE 4:**
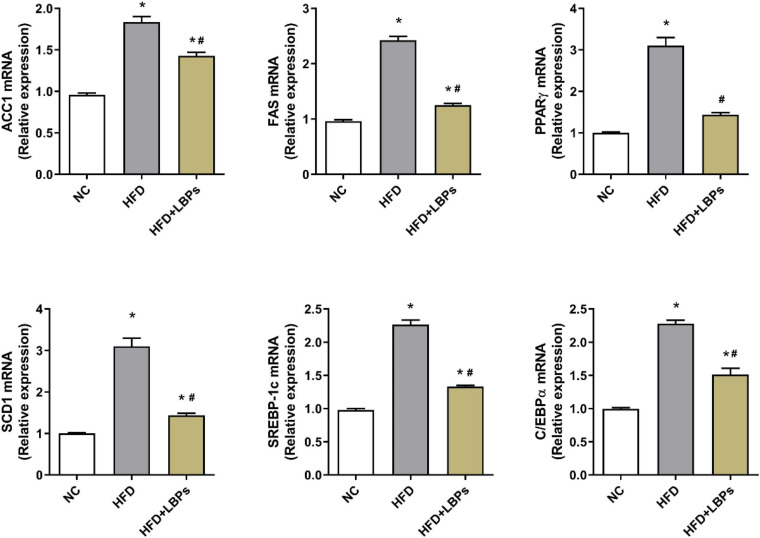
Effects of *Lycium barbarum* polysaccharides (LBPs) on expression of lipid-related genes (ACC1, FAS, PPARγ, SCD1, SREBP-1c, and C/EBPα) in epididymal adipose tissues in HFD-fed mice. *P < 0.05 vs. NC group and ^#^*P* < 0.05 vs. HFD group.

Administration of LBPs downregulated expression levels of adipogenesis-related gene including acetyl coenzyme A carboxylase 1 (ACC1), fatty acid synthase (FAS), synthesis *via* stearoyl-CoA desaturase 1 (SCD1), sterol regulatory element-binding protein 1c (SREBP-1c), peroxisome proliferator- activated receptor-γ (PPARγ), and CCAAT/enhancer-binding protein alpha (C/EBPα) in epididymal adipose tissue compared with HFD group (Figure 4) (*P* < 0.05).

### Gut Microbiota

**FIGURE 5:**
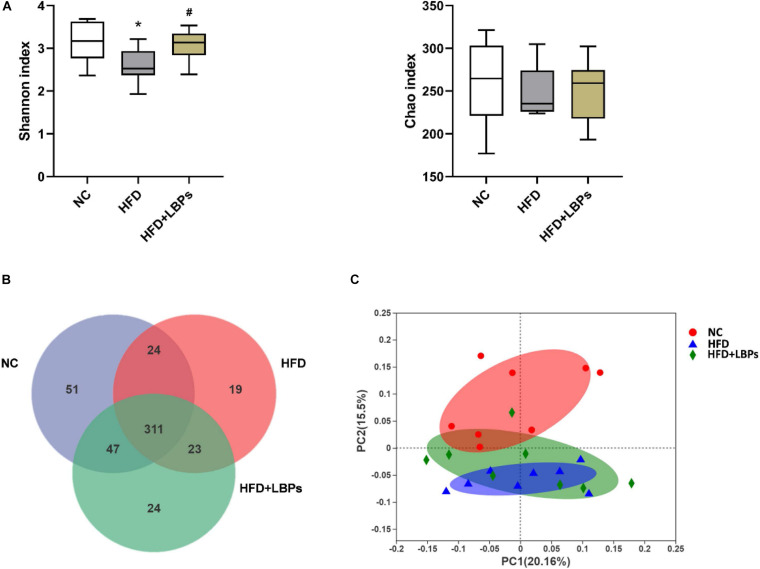
Effects of *Lycium barbarum* polysaccharides (LBPs) on diversity of modulation of gut microbiota. **(A)** Alpha diversity analysis, including Shannon, and Chao index. **(B)** Venn diagram of OTUs. **(C)** Principal coordinate analysis plot of gut microbiota based on unweighted unifrac metric. *P < 0.05 vs. NC group and ^#^*P* < 0.05 vs. HFD group.

Compared with the NC group, the Shannon index was decreased in the HFD, whereas the administration of LBPs reversed these indexes in HFD-fed mice (*P* < 0.05). LBP administration did not significantly influence the bacterial richness compared with the HFD group (Figure 5A) (*P* > 0.05). The analysis of OTU in the fecal showed that 335 OTUs were common between HFD + LBP and NC groups, whereas 42 OTUs were unique in the HFD group compared with 98 in the NC group. LBP administration increased the number of shared OTU from 335 to 358 (Figure 5B). For unweighted unifrac distance metrics, the NC group exhibited clustering of microbiota composition distinctly different from the HFD group. LBP treatment increased the similarity between the overall gut microbiota compositions of the HFD + LBP and NC groups, indicating that LBPs improved the structure of gut microbiota in HFD-fed mice (Figure 5C).

**FIGURE 6:**
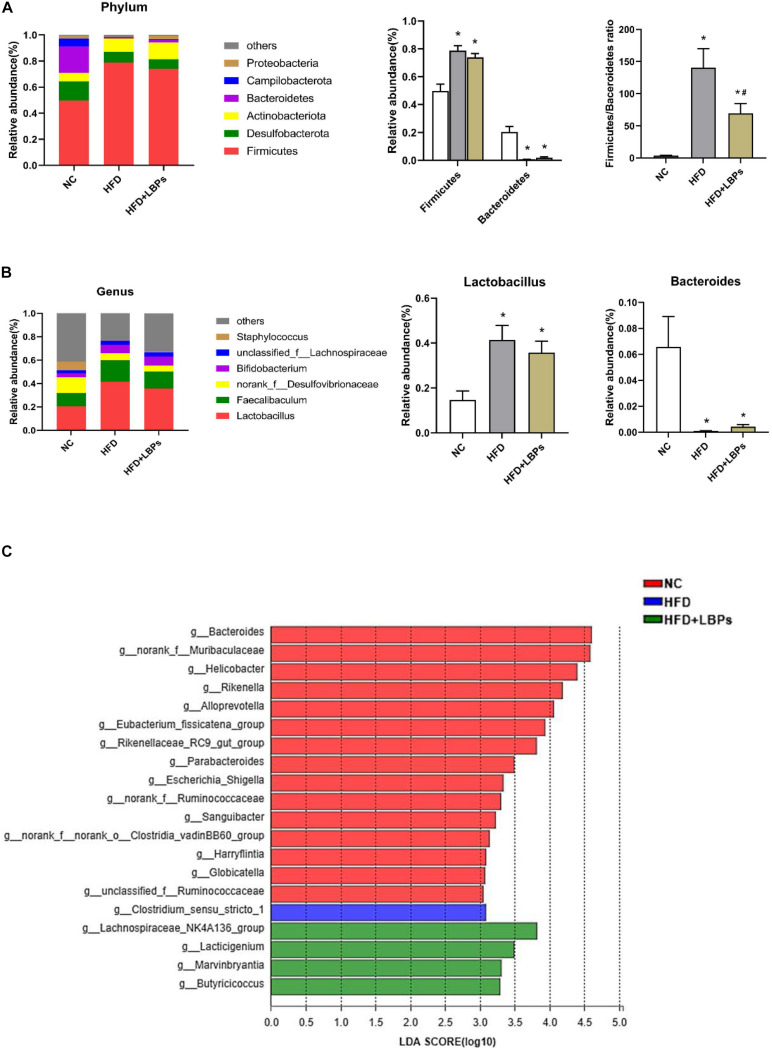
Effects of *Lycium barbarum* polysaccharides (LBPs) on composition of gut microbiota. **(A)** Composition of gut microbiota at phylum level and differences in relative abundance of *Firmicutes* and *Bacteroidetes* at phylum level. **(B)** Composition of gut microbiota at genus level and differences in relative abundance of *Lactobacillus*, and *Bacteroides*. LDA score plot with LDA scores (log 10) higher than 3 **(C)**. *P < 0.05 vs. NC group and ^#^*P* < 0.05 vs. HFD group.

At the phylum level, the top six phyla in the microbial communities included *Firmicutes*, *Desulfobacterota*, *Actinobacteriota*, *Bacteroidetes*, *Campilobacterota*, and *Proteobacteria* in the three groups, accounting for almost 99% of total bacteria. The HFD group reduced the relative abundance of *Bacteroidetes* and increased the relative abundance of *Firmicutes* (*P* < 0.05). After LBPs treatment, *Firmicutes* was reduced by 1.07-fold, whereas *Bacteroidetes* was increased by 2.73-fold in the HFD-fed mice compared with non-treatment mice. The *Firmicutes*/*Bacteroidetes* ratio was significantly increased by the HFD (*P* < 0.05). Differing from the HFD group, LBP administration significantly reduced the ratio of *Firmicute/Bacteroidetes* (Figure 6A) (*P* < 0.05). At the genus level, *Lactobacillus*, *Faecalibaculum*, *norank_f__Desulfovibrionaceae*, and *Bifidobacterium* were the dominant genera in the HFD + LBP group. HFD markedly increased the relative abundance of *Lactobacillus* and reduced the relative abundance of *Bacteroides* compared with the NC group (*P* < 0.05). After LBP treatment, the abundance of *Lactobacillus* and *Faecalibaculum* decreased by 1.16 and 1.27-fold, respectively, whereas *Bacteroides* was increased by 4.19-fold compared with the non-treatment group (Figure 6B). LDA effect size uses LDA to estimate the impact of abundance of each component on the different effects. Fifteen taxa were detected in the NC group. The *Bacteroides*, belonging to the phylum *Bacteroidetes*, produced a large effect on the dominant community, and was markedly enriched in the NC group. The HFD group was characterized by an increased amount of *Clostridium_sensu_stricto_1*, indicating a disruption of gut symbiosis. The genera of *Lachnospiraceae_NK4A136_group, Marvinbryantia*, *Butyricicoccus*, and *Lacticigenium* were the dominant phylotypes that contributed to the differences between the gut microbiota of HFD + LBP and HFD groups (Figure 6C).

### Short-Chain Fatty Acid Production in Colonic and Fecal Contents

**FIGURE 7:**
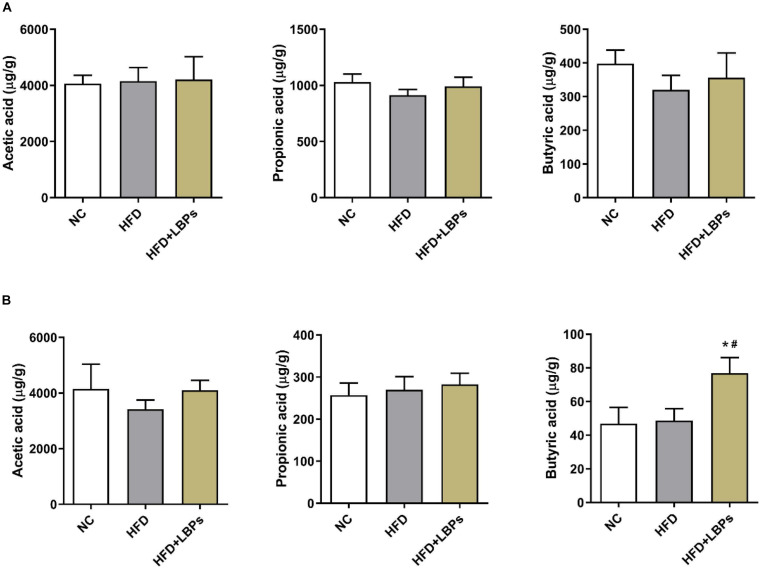
Effects of *Lycium barbarum* polysaccharides (LBPs) on SCFAs production in **(A)** colonic, and **(B)** fecal contents. *P < 0.05 vs. NC group and ^#^*P* < 0.05 vs. HFD group.

Colonic propionic acid and butyric acid concentrations in the former were reduced by 11.45 and 19.55%, respectively, in the HFD group compared with those in the NC group. LBP treatment increased the levels of colonic acetic acid (by 1.42%), propionic acid (by 8.67%), butyric acid (by 11.31%) and fecal acetic acid (by 19.80%), propionic acid (by 4.87%), and butyric acid (by 57.95%) compared with those in the HFD group (Figures 7A,B) (*P* > 0.05). The levels of fecal butyric acid were found to be higher in the HFD + LBPs group than in the HFD group (Figure 7B) (*P* < 0.05).

### Correlation Between the Gut Microbiota and Obesity-Related Parameters

**FIGURE 8:**
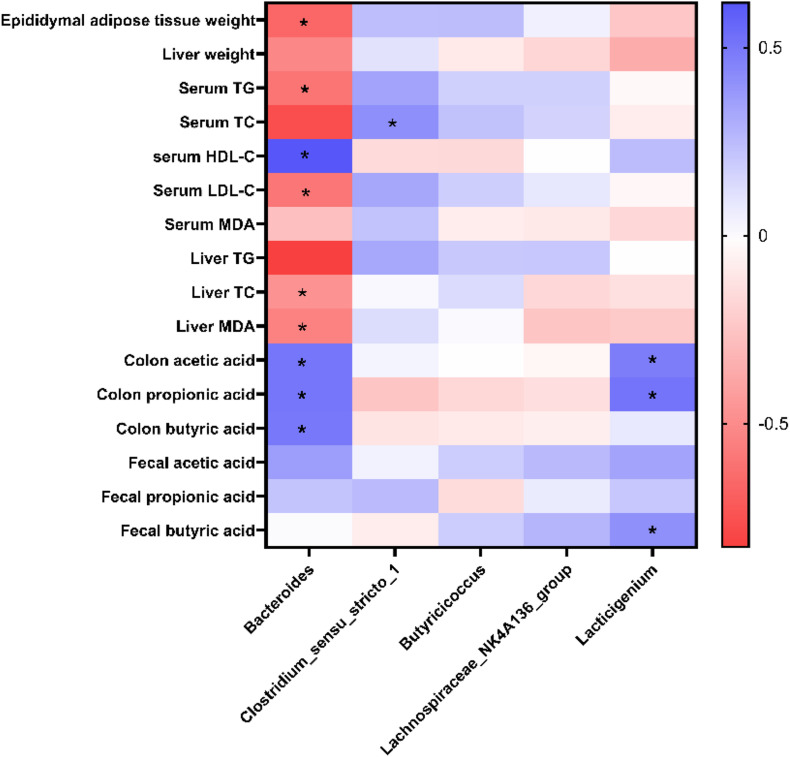
Heatmap of Spearman’s correlation between dominant genera and obesity-related parameters. *Significance was set at *P* < 0.05.

To further identify the potential correlation between gut microbiota and obesity-related parameters, a heatmap of Spearman’s correlation between the dominant genera, and obesity-related parameters was generated. A significant correlation was observed between the parameters and some specific taxa, such as *Bacteroides*, *Clostridium_sensu_stricto_1*, and *Lacticigenium* (*P* < 0.05). Among the specific genera, *Bacteroides* were negatively correlated with parameters such as epididymal adipose tissues weight, serum TG, serum LDL-C, liver TC, and liver MDA and positively correlated with serum HDL-C, colon acetic acid, colon propionic acid, and colon butyric acid. *Clostridium_sensu_stricto_1* was positively correlated with serum TC. *Lacticigenium* was positively correlated with SCFAs in the colon, suggesting it may be a probiotic that produces short-chain fatty acids (Figure 8).

## Discussion

Obesity is strongly associated with lipid metabolism, hepatic manifestation, metabolic abnormalities, and the composition of the gut microbiota (Parry and Hodson, 2017; Li et al., 2019; Zhi et al., 2019). LBPs have been reported to play an important role in anti-inflammatory (Wu et al., 2020), immunomodulatory (Zhu et al., 2020), and anti-obesity (Jia et al., 2016). We have previously shown that dietary LBP supplementation can improve intestinal microbial populations in early-weaned pigs (Chen et al., 2019). However, the effect of dietary LBPs on lipid metabolism and the modulation of gut microbiota have not been fully investigated in HFD-induced obese animal models. In the present study, we explored how the oral administration of LBPs regulates lipid metabolism *via* modulation in high-fat diet-fed mice.

In the current study, we found that HFD feeding increased the body weight, serum lipid profile, adipose tissue, and hepatic lipid accumulation in mice compared with the NC group for 10 weeks. These findings were consistent with some previous studies on the acceleration effect of HFD feeding on body weight and fat accumulation (Duan et al., 2019; Kong et al., 2019). We also found that LBP administration effectively alleviated HFD-induced dyslipidemia and hepatic lipid accumulation through decreasing TG, TC, LDL-C, and MDA in the serum and TG, TC, and MDA levels in the liver and increasing the serum HDL-C level in HFD feeding mice. These results are similar to the previous reports that found crude polysaccharide extracts (crude LBP), okra [*Abelmoschus esculentus* (L.) Moench] polysaccharides, *Grifola frondosa* polysaccharides, and *Cipangopaludina chinensis* polysaccharides reduced the level of serum lipids in the HFD model (Luo et al., 2004; Li et al., 2019; Liao et al., 2019; Xiong et al., 2019). The possible mechanism of LBP supplementation alleviating hepatic triglyceride production and accumulation is through upregulating lipolysis-degraded enzyme, and boosting fatty acid β-oxidation and inhibiting lipogenic enzyme production in the liver (Xiao et al., 2013; Jia et al., 2016). Furthermore, dysregulated lipid metabolism may induce lipid peroxidation, directly leading to oxidative stress (Xiao et al., 2014). We found that LBPs reduced the MDA levels in serum and liver. These hepatoprotective effects of LBPs were partly attributed to the activation of nuclear factor kappa B and the inhibition of the nucleotide-binding and oligomerization domain-like receptor protein 3/6 inflammasome pathway (Xiao et al., 2018). These data indicated that LBPs showed a vital role in alleviating HFD-induced anomalous changes of lipid profile and thus preventing lipid metabolic disorders.

Adipose tissue is known as an important energy reservoir and an essential regulator of energy homeostasis (Stolarczyk, 2017). Obesity is characterized by increased adipose tissue mass, which is caused by the increased number, and size of fat cells (Yang and Kim, 2015). Our results indicated that LBP treatment decreased the weight of adipose tissues and the size of adipocytes in epididymal adipose tissues in HFD-fed mice, which was consistent with the previous study that LBPs, and fermented *Momordica charantia* polysaccharides decreased fat accumulation in epididymal adipose tissues of HFD-fed mice (Zhao et al., 2016; Wen et al., 2019). Dysfunction and excessive accumulation of lipid in adipose tissue induce obesity, which is associated with atherosclerosis, cardiovascular diseases, dyslipidemia, and other metabolic syndromes (Moseti et al., 2016). Thus, reducing fat deposit and adipogenesis in the adipose tissue can prevent the development of obesity, and its associated diseases. Additionally, in the current study, LBP supplementation suppressed the upregulated expression level of ACC1, FAS, PPARγ, SCD1, SREBP-1c, and C/EBPα in adipose tissues of HFD-fed mice. PPARγ has been verified to be a ligand-activated transcription factor that can mediate the expression of fat-related genes and facilitate the process of adipogenesis (Lee et al., 2018). C/EBPα is considered an essential regulator that can induce adipocyte differentiation and adipogenesis through PPARγ (Lee et al., 2019). SREBP-1c can mediate the expression of fatty acid synthesis genes and activates lipogenic transcription factors such as ACC-1, FAS, and SCD1, which subsequently induces lipogenesis and the accumulation of lipid (Linden et al., 2018; Terzo et al., 2018). In addition, a recent study found that LBPs inhibited ACC and FAS expression by activating the SIRT1/adenosine monophosphate-activating protein kinase pathway and reducing lipid synthesis (Jia et al., 2016). It is worth noting that HFD feeding decreases PPARγ and C/EBPα expression, whereas *Polygonatum odoratum* polysaccharides increase PPARγ, and C/EBPα messenger RNA expression compared with that in HFD-fed mice (Wang Y. et al., 2018). The reason for inconsistent results may be partially lie in treatment conditions, diet ingredients, experimental duration, and species. Our findings fit well with previous studies reporting that *Gracilaria lemaneiformis* polysaccharides downregulate PPARγ and C/EBPα expression in the adipocyte tissues of HFD-fed mice (Sun et al., 2018). Therefore, the results showed that LBPs might be involved in decreasing fat adipogenesis and accumulation in epididymal adipose tissue mass by downregulating expression levels of adipogenesis-related genes.

The gut microbiota plays an important role in regulating energy homeostasis, glucose metabolism, and lipid metabolism in the host (Schoeler and Caesar, 2019). A variety of polysaccharides from plants have positive effects on modulating gut microbiota and preventing the development of obesity (Wang X. et al., 2018). Lower diversity of bacterial is associated with the probability of obesity and non-alcoholic fatty liver disease (Schwimmer et al., 2019; Chen et al., 2020). In our study, the administration of LBPs increased the diversity of bacteria by increasing the Shannon index, appearing to be a positive effect on the structure of the gut microbiota in obese mice. Numerous reports have shown that an increased *Firmicutes*/*Bacteroidetes* ratio promotes more lipid production and induces the development of abnormal weight gain, and chronic metabolic disease (Liu et al., 2019; Lu et al., 2019). Based on the heatmap of Spearman’s correlation, the relative abundance of *Bacteroides* was negatively associated with obesity cytokines, illustrating the potential ability to inhibit fat deposition in obese mice. In the present study, HFD consumption induced an increase in the *Firmicutes*/*Bacteroidetes* ratio, which is consistent with the previous study (Li J. et al., 2020). By contrast, oral administration of LBPs reduced the *Firmicutes*/*Bacteroidetes* ratio in HFD-fed mice, which might be a mechanism to explain the improvement of LBPs in HFD induced lipid accumulation in epididymal adipose tissues and liver. At the genus level, the HFD increased the proportions of *Clostridium_sensu_stricto_1*. LBP supplementation modulated gut microbiota and ameliorated intestinal dysbiosis by increasing the abundance of *Lachnospiraceae_NK4A136_group*, *Marvinbryantia*, *Butyricicoccus*, and *Lacticigenium* in HFD fed mice. *Clostridium_sensu_stricto_1* is generally perceived as pathogenic bacteria and interpreted as indicators of a less healthy microbiota (Huart et al., 2019; Shi et al., 2019). Some studies have reported that the proliferation of *Clostridium_sensu_stricto_1* was correlated with obesity, rheumatoid arthritis-associated atherosclerosis, dyslipidemia, and necrotic enteritis (Shi et al., 2019; Yang et al., 2019; Zeng et al., 2019). In the current study, the relative abundance of *Clostridium_sensu_stricto_1* was positively correlated with obesity, and the administration of LBPs decreased the relative abundance of *Clostridium_sensu_stricto_1*. The genus *Lachnospiraceae_NK4A136_group* was generally considered to be an SCFA producer, and its abundance was negatively correlated to inflammation (Wang J. et al., 2018). The genera *Marvinbryantia* are positively correlated to intestinal epithelial cell energy metabolism and butyrate production (Li A. L. et al., 2020). A butyrate-producing bacterium *Butyricicoccus* acts as a biomarker to predict obesity-related metabolic abnormalities, and the interventions of *Butyricicoccus* might be beneficial to weight loss and metabolic risk improvement (Luo et al., 2019; Zeng et al., 2019). It was reported that *Lacticigenium*, a lactic acid bacterium, produced acetic acids in addition to L-lactic acid (Iino et al., 2009). *Lacticigenium* showed a strong positive correlation with the produce of SCFAs. These studies support that supplementation of LBPs can promote the growth of beneficial bacteria and might contribute to improving gut dysbiosis induced by HFD.

Dietary polysaccharides can be fermented by gut microbiota provided with SCFAs, such as acetate, propionate, and butyrate (Shang et al., 2018). SCFAs are used as endogenous signaling molecules that could activate the G-protein-coupled receptor GPR43 associated with energy expenditure, leptin hormone secretion, and lipid metabolism (Hills et al., 2019; Schoeler and Caesar, 2019). Especially, butyric acid is able to mediate hepatic lipogenesis and fat oxidation (Li P. et al., 2020). In the present study, we found that LBPs increased the concentration of butyric acid in the feces of HFD-fed mice. Similar findings have been reported that some polysaccharides can increase SCFA production (Wang X. et al., 2019; Gu et al., 2020). Butyrate has the capacity to stimulate glucagon-like peptide-1 production and activate brown fat tissue, leading to sustained satiety and fat oxidation enhancement, thereby effectively preventing diet-induced obesity, insulin resistance, hypertriglyceridemia, and hepatic steatosis (Li et al., 2018; Vallianou et al., 2019). In particular, butyrate has been demonstrated to ameliorate insulin resistance and fatty acid oxidation, activate the adenosine monophosphate-activating protein kinase–acetyl–coenzyme A carboxylase pathway, and promote lipid metabolism (Mollica et al., 2017). Interestingly, some SCFA-producing intestinal microorganisms, such as *Lachnospiraceae_NK4A136_group* and *Lacticigenium*, and were enriched in the LBP-treated mice. All these results indicated that LBPs could increase SCFA production in the gut and thus benefit gut health, and prevent HFD-induced obesity.

## Conclusion

*Lycium barbarum* polysaccharide supplementation attenuated epididymal and liver fat accumulation and expression levels of adipogenesis genes in adipocytes. Furthermore, LBPs increased the relative abundance of SCFA-producing bacteria and increased SCFA production in HFD-induced mice. It implied that LBPs might be regarded as a potential functional food ingredient to prevent hyperlipidemia and modulate gut microbiota dysbiosis.

## Data Availability Statement

The datasets presented in this study can be found in online repositories. The names of the repository/repositories and accession number(s) can be found below: https://www.ncbi.nlm.nih.gov/bioproject/, PRJNA735319.

## Ethics Statement

The animal study was reviewed and approved by the Animal Care and Use Committee of the Hunan Agricultural University.

## Author Contributions

MY collected the data and drafted the manuscript. YxY and FW contributed to the animal sampling. HZ and XM performed the statistical analysis. YlY, BT, and JC provided resources and reviewed the manuscript. All authors contributed to the manuscript revision and approved the submitted version.

## Conflict of Interest

The authors declare that the research was conducted in the absence of any commercial or financial relationships that could be construed as a potential conflict of interest.

## Publisher’s Note

All claims expressed in this article are solely those of the authors and do not necessarily represent those of their affiliated organizations, or those of the publisher, the editors and the reviewers. Any product that may be evaluated in this article, or claim that may be made by its manufacturer, is not guaranteed or endorsed by the publisher.

## Footnotes

1
http://drive5.com/usearch/

2
http://sourceforge.net/projects/rdp-classifier/

3
https://www.arb-silva.de/

4
https://www.mothur.org/wiki/Download_mothur
