# Supplementary material for: Supplementation With Lycium barbarum Polysaccharides Reduce Obesity in High-Fat Diet-Fed Mice by Modulation of Gut Microbiota
Source: Front Microbiol. 2021 Aug 26;12:719967. doi: 10.3389/fmicb.2021.719967 (PMC8427603; doi:10.3389/fmicb.2021.719967)
Supplement: Supplementary file 1 [file Table_1.DOCX]

| Genes | Primer sequences | Gene bank |
| --- | --- | --- |
| ACC1 | F: 5′-GGGCTACCTCTAATGGTCTT-3′ | NM_133360 |
|  | R: 5′-CTACCTGATGGTAAATGGGA-3′ |  |
| FAS | F: 5′-CTTGGGTGCTGACTACAACC-3′ | NM_007988 |
|  | R: 5′-GCCCTCCCGTACACTCACTC-3′ |  |
| PPARγ | F: 5′- ATTCTGGCCCACCAACTTCGG -3′ | NM_013124 |
|  | R: 5′- TGGAAGCCTGATGCTTTATCCCCA -3′ |  |
| SCD1 | F: 5′- CGGAAATGAACGAGAGAAGG -3′ | NM_009127.4 |
|  | R: 5′- CCGAAGAGGCAGGTGTAGAG -3′ |  |
| SREBP-1c | F: 5′- CTTCTGGAGACATCGCAAAC -3′ | BC056922 |
|  | R: 5′- GGTAGACAACAGCCGCATC -3′ |  |
| C/EBPα | F: 5′-AGACATCAGCGCCTACATCG-3′ | NM_007678 |
|  | R: 5′-TGCAGGTGCATGGTGGTC-3′ |  |
| β-actin | F: 5′- TGGAATCCTGTGGCATCCATGAAA-3′ | NM_007393.5 |
|  | R: 5′- TAAAACGCAGCTCAGTAACAGTCCG-3′ |  |

**Table S1** Sequences of primers used for quantitative real-time PCR assays.
